# Supplementary material for: Hybrid Models and Biological Model Reduction with PyDSTool
Source: PLoS Comput Biol. 2012 Aug 9;8(8):e1002628. doi: 10.1371/journal.pcbi.1002628 (PMC3415397; doi:10.1371/journal.pcbi.1002628)
Supplement: Text S4 — Complete source code for the PyDSTool package (version 0.88.120504). Includes API documentation and help files linking to web pages. This file is identical to the current public release on Sourceforge.net. (ZIP) [file pcbi.1002628.s004.zip › PyDSTool/html/PyDSTool.Toolbox.optimizers.line_search.simple_line_search-module.html]

xml version="1.0" encoding="ascii"?


PyDSTool.Toolbox.optimizers.line\_search.simple\_line\_search


| Home | Trees | Indices | Help | PyDSTool |
| --- | --- | --- | --- | --- |

Package PyDSTool ::
Package Toolbox ::
Package optimizers ::
Package line\_search ::
Module simple\_line\_search

[hide private]

[frames] | no frames]

# Module simple\_line\_search

source code

A simple line search, in fact no searches at all


|  |  |
| --- | --- |
| Classes | [hide private] |

SimpleLineSearch  
A simple line search, takes a point, adds a step and returns it

| Home | Trees | Indices | Help | PyDSTool |
| --- | --- | --- | --- | --- |

|  |  |
| --- | --- |
| Generated by Epydoc 3.0.1 on Fri May 4 15:24:05 2012 | http://epydoc.sourceforge.net |
